# Left Ventricular Outflow Tract Obstruction in Patients Treated With Milrinone for Cerebral Vasospasm: Case Report and Literature Review

**DOI:** 10.2196/31019

**Published:** 2022-04-11

**Authors:** Charles Baulier, Marc Lessert, Jean-Louis Chauvet, Pauline Garel, Alexandre Bergis, Julie Burdeau, Thomas Clavier

**Affiliations:** 1 Anesthesia and Intensive Care Department Rouen University Hospital Rouen France; 2 Intensive Care Unit Elbeuf General Hospital Elbeuf France; 3 Cardiology Department Rouen University Hospital Rouen France

**Keywords:** ventricular outflow obstruction, subarachnoid hemorrhage, vasospasm, intracranial, milrinone, hemorrhage, neurosurgery, neurology, surgery, pharmaceutical

## Abstract

Subarachnoid hemorrhage is associated with high morbidity and mortality, and cerebral arterial vasospasm is one of its main complications that determines neurological prognosis. The use of intravenous milrinone is becoming more common in the treatment of vasospasm. This molecule has positive inotropic and vasodilating properties by inhibiting phosphodiesterase-3. Its most described side effects are cardiac arrhythmias and arterial hypotension. In this paper, we raise a new issue concerning milrinone and discuss an undescribed side effect of this treatment, left ventricular outflow tract obstruction (LVOTO). Dynamic LVOTO is a clinical situation favored by hypovolemia, decreased left ventricular afterload, and excessive inotropism that can lead to severe hemodynamic failure and pulmonary edema. To our knowledge, this is the first study describing milrinone-induced LVOTO. This could compromise cerebral perfusion and therefore the neurological prognosis of patients. While it is known that catecholamines may induce LVOTO, milrinone-induced LVOTO appears to be a new pathophysiological entity of which neurosurgical intensivists should be aware.

## Introduction

Subarachnoid hemorrhage (SAH) by aneurysmal rupture accounts for 1% to 5% of all strokes [1,2]. One of the main complications conditioning SAH prognosis is cerebral arterial vasospasm, which occurs in 50% to 70% of cases [3]. Cerebral vasospasm is mostly asymptomatic, but may be complicated by secondary ischemic neurological deficit. Early management of asymptomatic forms of vasospasm can prevent ischemic complications and improve functional prognosis [4]. The first-line prophylactic treatment for cerebral vasospasm is nimodipine [5]. However, cerebral vasospasm can occur despite this prophylactic treatment, and several curative treatments have been proposed. Some of them, such as papaverine or milrinone, are administered directly in the vessel during cerebral arteriography [6,7]. Intravenously administered molecules are also proposed in this indication such as sildenafil, magnesium sulfate, or milrinone [8-12]. Milrinone appears to be a promising treatment to alleviate cerebral vasospasm [11]. It is a phosphodiesterase-3 inhibitor, with positive inotropic effect and systemic arterial vasodilator effect, whose main therapeutic indication is cardiogenic shock [13]. However, milrinone can induce cardiac arrhythmias by inhibiting the degradation of cyclic adenosine monophosphate, which leads to an increase in intracellular calcium.

In this paper, we raise new issues concerning milrinone and report an undescribed side effect of this treatment that we observed in two patients hospitalized in a neurosurgical intensive care unit (ICU) for SAH (with two different clinical presentations): dynamic left ventricular outflow tract obstruction (LVOTO). This is characterized by a pressure gradient between the left ventricle and the aorta. The narrowed left ventricular outflow tract is the site of substantial flow, which can lead to anterior translation of the mitral valve (systolic anterior motion [SAM]) and regurgitation of the valve. Fixed LVOTO is due to particular anatomical conditions that can be corrected by surgery. Dynamic LVOTO is favored by the addition of predisposing factors (decreased preload and afterload, increased inotropism). Clinically, LVOTO may induce hemodynamic failure up to shock, and neurosurgical intensivists should be aware of this complication of milrinone infusion.

## Clinical Presentations of Left Ventricular Outflow Tract Obstruction Induced by Milrinone

To our knowledge, we describe here the two first cases of LVOTO induced by milrinone.

### Ethical Considerations

Informed consent was obtained from all individual patients described in this paper.

This study has been performed in accordance with the ethical standards laid down in the 1964 Declaration of Helsinki. The Comité d'Ethique pour la Recherche sur Données Existantes et/ou hors loi Jardé (CERDE-HLJ) of the Rouen University Hospital provided the ethical approval (E2021-91).

### Acute and Severe Left Ventricular Outflow Tract Obstruction Induced by Milrinone

A 48-year-old male was admitted in a neurosurgical ICU for a Fisher 4 and World Federation of Neurosurgical Societies (WFNS) 1 SAH, by rupture of a right terminal and internal carotid aneurysm. His medical history included arterial hypertension, essential tremors, and active smoking. He had no cardiological follow-up and was not taking long-term medication. Cardiac auscultation was normal on admission. An emergency external ventricular drainage was performed to treat acute hydrocephalus followed by a radioembolization of the two aneurysms 24 hours after admission. Prophylactic treatment with nimodipine was administered orally. Faced with an unfavorable neurological evolution on the third day, with the appearance of aphasia and right hemineglect, another angio–computerized tomography (CT) was performed, showing a diffuse vasospasm. A treatment with intravenous milrinone was started, initially at an infusion rate of 0.5 μg per kg per minute and increased 2 hours later to 1 μg per kg per minute because of good hemodynamic tolerance. The introduction of milrinone was followed by a partial regression of aphasia, and nimodipine was maintained.

Due to the reappearance of complete aphasia 7 days after ICU admission, the patient benefited from in situ dilatations of the anterior and posterior cerebral arteries for refractory vasospasm. This procedure was followed by a complete regression of neurological symptoms.

**Figure 1:**
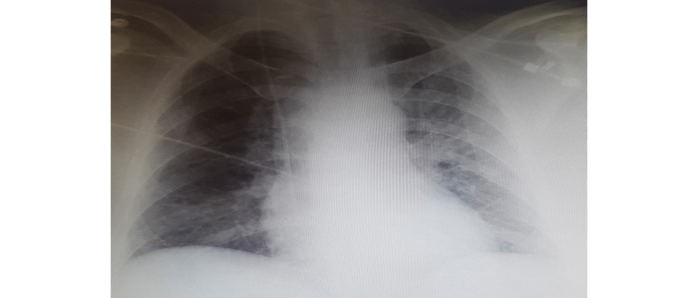
Thoracic radiography of the first patient. Mitral regurgitation associated with left ventricular outflow tract obstruction is most often eccentric and travels to the left pulmonary veins, resulting in unilateral acute pulmonary edema in this patient.

Two weeks after ICU admission, while the patient was awake, extubated, apyretic, and hemodynamically stable without catecholamines, he presented a brutal hemodynamic failure with a systolic blood pressure inferior to 40 mmHg, with 120 bpm tachycardia and arterial oxygen desaturation up to 75%. A previously unknown systolic heart murmur was found. The patient remained fully conscious during this episode. He benefited from fluid therapy by 2 liters of crystalloids, orotracheal intubation due to respiratory failure, and norepinephrine infusion, which was rapidly increased at 2 μg per kg per minute. A transthoracic echocardiography was performed and showed an intraventricular gradient of obstruction, a hypercontractile left ventricle with telesystolic exclusion, and a grade 4 mitral regurgitation with SAM of the mitral valve. The interventricular septum thickness was normal, excluding ventricular hypertrophy, and cardiac ventricles were not dilated. Chest X-ray showed unilateral alveolar opacity of the left lung, consistent with an acute pulmonary edema (Figure 1). A chest CT scan did not find pulmonary embolism or aortic dissection. The situation improved within 3 hours of milrinone stopping, allowing a decrease in the doses of norepinephrine and a normalization of cardiac auscultation. Although milrinone was administered at a constant dosage of 1 µg per kg per minute, the clinical presentation led to finding the origin of the shock: an accidental bolus of milrinone. The main hypothesis is that the catheter plicated when the patient was placed in a sitting position, the electric syringe did not stop, and the bolus of milrinone occurred when the obstruction was removed. The patient was extubated the following day, and norepinephrine was definitively stopped 2 days after the accident. The control echocardiography performed 2 days after the shock showed a complete regression of LVOTO, SAM, and mitral insufficiency. The neurological outcome was finally favorable with hospital discharge 1 month after ICU admission.

### Subacute and Moderate Left Ventricular Outflow Tract Obstruction Induced by Milrinone

A 69-year-old patient was admitted in a neurosurgical ICU for a Fischer 4 and WFNS 4 SAH secondary to a right giant carotid and ophthalmic aneurysm rupture. Her medical history included hypothyroidism and osteoporosis. She had no history of heart disease and had no regular cardiac follow-up. Cardiac auscultation was normal at admission. Neurological management was marked by an emergency external ventricular drainage to treat acute hydrocephalus, followed by radioembolization of the aneurysm and the initiation of nimodipine treatment. A follow-up cerebral angio-CT performed 7 days after ICU admission revealed diffuse cerebral vasospasm, which prompted the introduction of milrinone (0.5 μg/kg/min, then increased to 1 μg/kg/min because of good hemodynamic tolerance). One day later, the patient was febrile with an inflammatory syndrome leading to the diagnosis of a *Staphylococcus epidermidis* meningitis on ventricular drain. Antibiotic therapy by linezolide was therefore started, and a change of ventricular drain was performed 48 hours after the initiation of antibiotic therapy.

**Figure 2:**
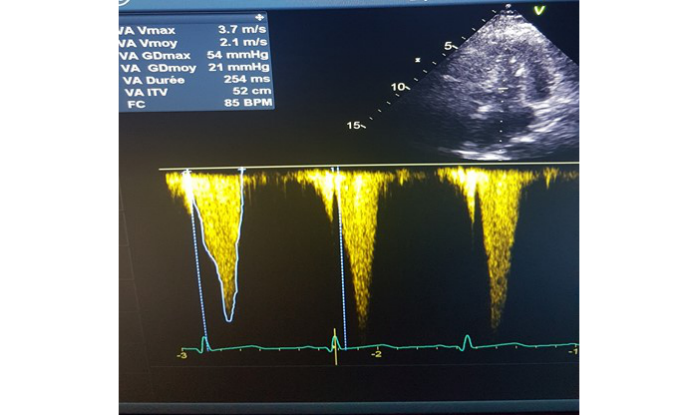
Apical 5 cavities view of transthoracic echocardiography. Diagnostic of left ventricular outflow tract obstruction is assessed here by a maximal pressure gradient measured at 54 mmHg. Velocity peak is maximum in telesystolic.

A systolic murmur was reported 3 days after milrinone initiation. It was midsystolic, heard in the second right intercostal space, intense, and radiating to the carotid arteries, strongly suggestive of aortic stenosis. The patient presented a stable hemodynamic state but a Glasgow Coma Score fluctuating between 12 and 15. Transthoracic echocardiography was performed at the onset of this heart murmur to look for a valvulopathy. The echocardiography revealed a gradient of left intraventricular obstruction with a maximum telesystolic peak measured at 54 mmHg (Figure 2) and a hypercontractile left ventricle with telesystolic exclusion. Left ventricle filling pressures were estimated to be low (E/A ratio equal to 0.7). The interventricular septum thickness was normal. Within 24 hours after the exam, an arterial hypotension appeared, which resolved after fluid therapy by 1 L of crystalloids. In view of the hemodynamic improvement and the good neurological course, treatment with milrinone was continued at the same dose, and there was no follow-up echocardiography before stopping the treatment. Milrinone and nimodipine were stopped 21 days after the onset of SAH, allowing ICU discharge the next day. At ICU discharge, the patient no longer had systolic murmur, and the echocardiography showed no intraventricular obstruction gradient nor telesystolic exclusion of the left ventricle. Finally, the patient was discharged without neurological deficit.

## Pathophysiology of Left Ventricular Outflow Tract Obstruction

The dynamic LVOTO phenomenon is secondary to a decreased preload and a decreased postload with increased inotropism thus reducing the telediastolic and telesystolic volumes of the left ventricle [14-16]. These conditions often occur in common situations in the ICU, such as the use of positive inotropes in hypovolemic patients, or during septic shock [17]. They are also described in postoperative mitral or aortic valve replacement, in intraoperative noncardiac surgery, or in patients with hemorrhagic shock [16]. They result in a reduction of the size of the left ventricle in systole and in the development of an obstruction that may be medio-ventricular or caused by a SAM. Both types of obstruction, combined or not, are possible [17]. In case of SAM, the attraction of the large mitral valve toward the interventricular septum creates an opening of the valve that leads to functional mitral regurgitation, which can be important as in our first case. This mitral insufficiency disappears after SAM correction.

The difference must be made between fixed LVOTO, due to underlying anatomical factors (excessive mitral tissue, mitro-aortic angle less than 120°, septal hypertrophy) that are sometimes correctable by surgery. In patients with hypertrophic obstructive cardiomyopathy (HOCM), the onset or aggravation of the stenosis may be seen during hypovolemia, vasodilatation, physical exertion, or dobutamine stress ultrasound [16-18]. In patients who are HOCM free in the ICU, obstruction is found in the presence of a classic triad: hyperkinetic left ventricle, tachycardia, and hypovolemia [19]. Thus, logically, catecholamines are described as a potential inducer of LVOTO. This has mostly been described with inotropes such as dobutamine but also with dopamine and norepinephrine [20-25].

## Diagnosis of Left Ventricular Outflow Tract Obstruction

Diagnosis of LVOTO is difficult and can only be done with echocardiography. The diagnosis relies on the presence of an intraventricular pressure gradient (measured in continuous Doppler) with a velocity peak ≥1 m/s (or LVOTO pressure gradient at least 30 mmHg) or an anterior systolic movement of the large mitral valve, which is attracted into the left ventricle flushing chamber (SAM) [16,17]. A meso- or telesystolic exclusion of the middle part of the left ventricle may also be seen. This entity differs from fixed LVOTO because it occurs on a healthy heart, with a normal thickness of the interventricular septum.

The main differential diagnosis in this context of SAH is neurogenic myocardial stunning. This occurs in the acute phase of SAH. Unlike LVOTO, beta receptor overstimulation results in myocardial sideration. The common feature of the two conditions is the worsening with the use of catecholamines.

## Imputability of Milrinone in the Occurrence of Left Ventricular Outflow Tract Obstruction

We did not find any study describing milrinone-induced dynamic LVOTO. When looking for studies dealing with hemodynamic effects of milrinone, several of them have described a decrease in systemic and pulmonary vascular resistance, an increase in cardiac index, an improvement in diastolic function, and a decrease in pulmonary capillary wedge pressure [26-29]. Other side effects frequently reported are headache, tachycardia, arterial hypotension, arrhythmias (especially atrial fibrillation), and ventricular extrasystoles [7,26-29]. These complications occur more frequently in patients with impaired left ventricular ejection fraction.

However, our cases showed the occurrence of LVOTO after milrinone infusion start in two patients without any cardiac disease before ICU admission. Indeed, in our first case, the only predisposing factor appeared to be a punctual bolus of milrinone, resulting in acute LVOTO with SAM, functional mitral insufficiency, and shock. Mitral regurgitation associated with LVOTO is most often eccentric and travels to the left pulmonary veins [30], resulting in unilateral acute pulmonary edema in this patient. In our second case, the hemodynamic effects of milrinone (afterload decrease and increased inotropism) were added to a relative decrease in preload induced by sepsis. The consequence was then a symptomatic subacute and less severe form of LVOTO clinically translated by the appearance of a new heart murmur and arterial hypotension requiring fluid therapy. The systolic murmur persisted after punctual use of crystalloids, and only disappeared when the milrinone infusion was stopped. These elements support, in our opinion, the hypothesis that the use of milrinone is the main and triggering mechanism in this case, sepsis being only an aggravating factor.

Given our observations and on the basis of LVOTO pathophysiology, we think that milrinone, by combining a positive inotropic effect with a systemic arterial vasodilator effect, could be a molecule that strongly promotes the occurrence of LVOTO.

## Implication of Milrinone-Induced Left Ventricular Outflow Tract Obstruction for Neurosurgical Intensivists

Milrinone plays an important role in the treatment of cerebral vasospasm, and several neurosurgical ICU teams are using it for their patients with SAH. However, our reports and the analysis of the LVOTO pathophysiology suggest that in patients that are hypovolemic or septic, milrinone should probably be used with caution. The onset of LVOTO due to milrinone may be difficult to diagnose because of the lack of a specific clinical sign. However, its consequences are potentially serious since the occurrence of shock could worsen cerebral ischemia and neurological prognosis in patients already treated for arterial vasospasm. Echocardiography should probably be proposed early in patients with hemodynamic instability associated with milrinone. The use of echocardiography should probably be proposed in the presence of the aforementioned triggers or in case of hemodynamic instability in patients treated with milrinone. Prospective studies on a larger patient population are needed to determine the incidence of these disorders and their impact on the prognosis of patients treated for arterial vasospasm with milrinone.

## Treatment of Left Ventricular Outflow Tract Obstruction Induced by Milrinone

In patients with septic shock, LVOTO appears to be an independent risk factor for mortality [17]. It therefore makes sense to try to reduce or even eliminate this complication among patients in the ICU. The treatment is mostly based on the management of the triggering factors such as the correction of a hypovolemia, the stop of inotropic treatments, and the treatment of a sepsis. The occurrence of tachycardia, which is frequent in neurosurgical ICU, should be carefully monitored, as it aggravates the dynamic LVOTO. In some cases, the use of α-agonists vasoconstrictors or the introduction of a beta-blocker to decrease the ventricular pressure gradient may be proposed [19].

## Conclusion

LVOTO is probably an underestimated clinical situation in neurosurgical ICU. It may occur in patients without any previous cardiac diseases, and its diagnosis relies on echocardiography. There are numerous triggering factors that are frequent in patients in the ICU and that cumulatively promote its occurrence. Milrinone, because of its positive inotropic effect and systemic arterial vasodilator effect, appears to be a molecule that may provide LVOTO. Further studies are needed to evaluate the incidence of dynamic LVOTO and its impact on the prognosis of patients with cerebral vasospasm treated with milrinone.

## Abbreviations

CT: computerized tomography
HOCM: hypertrophic obstructive cardiomyopathy
ICU: intensive care unit
LVOTO: left ventricular outflow tract obstruction
SAH: subarachnoid hemorrhage
SAM: systolic anterior motion
WFNS: World Federation of Neurosurgical Societies

## Data Availability

The raw data supporting the observations reported in this manuscript can be made available on request by the authors to any qualified researcher.
